# Rheumatic Fever Treated by Acetate of Potash

**Published:** 1851-06

**Authors:** 


*Rheumatic Fever treated by Acetate of Potash.*—(Under the care of Dr. Golding Bird.) One of the acute diseases which the hospital physician is most frequently called upon to treat is rheumatic fever; we have ample opportunities of seeing this fact verified, and have taken advantage of that circumstance to observe the methods of treatment which are employed in the hospitals of London against this very common affection. If our readers will refer to previous *Lancets*, they will find acute rheumatism treated with colchicum, acetate and nitrate of potash, and lemon-juice respectively. We now beg their attention for a few moments, whilst we give an outline of Dr. Golding

Bird's treatment of rheumatic fever with acetate of potash alone. It is of course understood that the generality of physicians abstract blood, either generally or locally, and give mercury, when symptoms of pericarditis become manifest, these measures being adopted independently of the peculiar line of treatmedt.

No one will be surprised when we state that we were pecnliarly interested in the progress of the cases for which Dr. Bird prescribed acetate of potash ; for his lectures, delivered in 1848 at the College of Physicians, had taught the profession *why* the duretic salt above mentioned might be useful in rheumatism. (Lectures on the Influence of Researches in Organic Chemistry on Therapeutics, in Relation to the depuration of the Blood, *Medical Gazette*, 1848, vols. I. and II.) Dr. Golding Bird, after giving a detail of the solid matters found in the urine whilst the functions are in a normal state, adds: "but we also know that the researches of Wohler have shown us something more—viz,, that whatever substances exist dissolved in the blood not necessary or fit for the repair of the structure of our frame, invariably escape from the body by the kidneys. The injection of saline bodies, coloring matter, &c., readily proves Wohler's statement." The lecturer mentions, a little further on, that the septic agents do not re-appear in their original form, but that they undergo metamorphosis ; and proceeds in these terms :—"Can we at will by therapeutic agents produce this depurating effect, and by hastening the metamorphosis of matter, aid the removal of a *materies morbi*, whether itself the exciting cause or effect of antecedent morbid action?"

Before we state which are the *therapeutic agents* most likely thus to depurate the blood, we should mention what efficacy Dr. Bird ascribes to the increase in the secretion of the the kidneys :—"1t will be found that the increase of water does not escape alone, but there is really washed away with it a certain although not very large quantity of solid debris." "This is a key to the cures effected by mineral springs." "I would press upon the practitioner the importance of directing his attention to diuretics, not as merely helping the pumping off of the water, but as *renal alteratives*, as remedies aiding the removal from the body of injurious matter." The diuretic to which Dr.

Golding Bird gives the preference for removing the *materies morbi* in rheumatism is acetate of potash ; it appears that this agent has been extensively used by Dr. Bird. We shall just give the outline of two cases, the progress of which we very lately watched with great care.

*Case* 1.—Notes taken by Mr. G. Evans, clinical clerk. Jane P., aged sixteen years, a girl, of sanguineous temperament, following the occupation of nursery-maid, was admitted under the care of Dr. Golding Bird, on December 18, 1850. The patient's parents and sisters reside in Gloucestershire, and all enjoy good health ; she states that while in service (which she left the evening before admission) she slept in a kitchen underground, this place being, according to her statement, tolerably dry, but deficient in cleanlinss. Her health had always been good up to a fortnight before she came into the hospital, when she was attacked with dull, aching pain in her right ankle ; the joint became stiff and swollen, and the knee was soon similarly effected in spite of the treatment devised by her *mistress.* She was at length placed under medical care, but the malady made rapid progress, and had soon invaded the left ankle and knee, as well as the shoulders.

She was thus laboring under inflammation of the principal joints when admitted, at which period she presented the following symptoms :—skin hot and dry ; tongue slightly furred ; pulse quick and full, and slight headache. The wrists, knees, and ankles, swollen, stiff, and painful, and yielding a dry, rubbing sound on being moved. Bowels and catamenia regular ; appetite tolerable ; sleep very indifferent. Dr. Bird ordered twenty-five grains of acetate of potash to be taken every fourth hour in camphor mixture, and eight grains of Dover's powder at night. The patient was much better on the third day ; she moved her wrists with facility, and without pain ; the skin had become moist ; she slept better ; the bowels acted once a day, and the appetite was improved. Pulse the same as before, and tongue more furred. The medicine was continued, and on the seventh day the ankles were free from pain, though the arms became somewhat stiff and swollen; all the other symptoms improving.

On the fourteenth day, the pulse was natural, and the patient quite free from pain in any joint, the appetite was very much improved: and to each dose of the acetate of potash hitherto regularly taken every fourth hour, four grains of ammonio-citrate of iron were added, and the girl was desired to get up for two or three hours a day. The latter injunction was carried out to a greater extent than had been prescribed, and the cousequence was a slight relapse on the seventeenth day; this, however, soon gave way, and on the twenty-first day the patient was discharged quite well.

*Case* 2.—Notes taken by Mr. G. Puckle, clinical clerk. Ellen G., aged twenty-nine years, a married woman, who has had one child, and is between three and four months advanced in pregnancy, was admitted under the care of Dr. Golding Bird, Dec. 18, 1850. (It will be perceived that both patients, in Cases 1 and 2, were admitted on the same day, and as they lay in the same ward, it was very interesting to watch the effect of the acetate of potash upon these patients.) This woman is a native of Ireland, of florid complexion, irritable and sanguineous temperament; she has usually enjoyed good health, and is affected with rheumatism for the first time. Five days before her coming into the hospital, the patient got very wet, and the same evening the right wrist became painful and swollen, the next day the knee, and subsequently the shoulder of the same side became similarly affected.

On admission, the right shoulder, wrist, and knee, were exquisitely tender, as well as swollen and painful; the cheeks were flushed; there was headach, and sour-smelling perspiration; tongue covered with a dirty-white fur, and red at the tip and edges; pulse quick, full and bounding; urine turbid and acid, great restlessness, and no sleep for several nights. Dr. Bird ordered half a drachm of acetate of potash to be taken every fourth hour, in camphor mixture; and ten grains of Dover's powder at night. The doses were somewhat larger than in the preceding case, both probably on account of the difference of age and the severity of the symptoms. As the joints were exquisitely tender, soda poultices (the ordinary linseed poultice, made with a solution of carbonate of soda, instead of plain water) were applied to the affected articulations. On the third day, the patient was considerably relieved. the bowels were, however, confined. Calomel and colocynth were ordered, and the acetate continued

The improvement was steady and gradual; and on the sixteenth day all the joints were well, with the exception of a little stiffness about the shoulder. The patient then took two grains of sulphate of quinine three times a day, and was soon quite well. The urine was in both cases considerably increased in quantity.

It will now be necessary to inquire for what reason Dr. Bird prefers the acetate of potash to other diuretics. If we turn to the above-mentioned Lectures, we find the following explanation :—

"Squill, copaiba, broom, juniper, &c.,merely increase the fluids, and are useful in dropsy." "Remedies which exert no chemical action on organic matter out of the body, appear to be incapable of augmenting the quantity of solids in the urine, and hence are of use only in the elimination of water; they may and do act as renal hydragogues, but not as renal depurants." "We have next to notice those remedies among the reputed diuretics which increase the metamorphosis of tissue, and act as *depurating agents.* This class includes the alkalies, their carbonates, and their salts, with such acids as in the animal economy are capable of being converted into carbonic acid, including the acetates, tartrates, and citrates of soda and potash. These remedies act all alike—they all actively stimulate the functions of the kidneys, and increase the bulk of urine ; but they do more—they actually increase the metamorphosis of tissue by, in all probability, a direct chemical action on the elements of worn-out and exhausted tissue or other matter in the capillary laboratory of the body."

As to nitrate of potash, Dr. Bird has found it inferior to the acetate of the same alkali. He has used the latter with much advantage, as he finds that " the quantity of fluid removed from the body by the nitrate of potash is far less than that which is carried off under the solvent influence of those agents which act more energetically on animal matter."

